# Environmental Assessment Strategies for Biodegradable Polymer Composites: A Review of Life Cycle Perspectives on Agro-Waste Reinforced Materials

**DOI:** 10.3390/polym18060700

**Published:** 2026-03-13

**Authors:** Kastytis Pamakštys, Anastasiia Sholokhova, Inga Gurauskienė, Visvaldas Varžinskas

**Affiliations:** 1 Center for Research on Advanced Packaging Materials and Technologies, Kaunas University of Technology, 51424 Kaunas, Lithuania; kastytis.pamakstys@ktu.lt (K.P.); visvaldas.varzinskas@ktu.lt (V.V.); 2 Institute of Environmental Engineering, Kaunas University of Technology, 44242 Kaunas, Lithuania; inga.gurauskiene@ktu.lt

**Keywords:** biocomposites, life cycle assessment, sustainability, environmental impacts, natural fibres, composite materials, agro-waste

## Abstract

The growing interest in bio-based and biodegradable polymer composites reinforced with agricultural waste reflects global efforts to reduce dependence on fossil resources and improve the sustainability of materials. However, biocomposites are not necessarily more sustainable, and their environmental performance requires careful life cycle assessment (LCA). This review critically analyses recent LCA studies of biodegradable biocomposites reinforced with agricultural waste, focusing on methodological choices, data quality, results and limitations. A systematic literature review was conducted using the Scopus database, focusing on studies from the last five years. Selected studies were examined using a structure consistent with ISO 14040, with defined data extraction categories and key questions. The analysis shows that although biocomposites often demonstrate advantages in terms of climate change and fossil resource depletion compared to traditional materials, the results vary significantly depending on the definition of the functional unit, geographical context, processing pathways, and data assumptions. Limitations include reliance on laboratory data, uncertainties, incomplete system boundaries, inconsistent allocation methods, and limited end-of-life (EoL) modelling. Overall, the review highlights the need for improved data quality, performance-based functional units, geographically representative inventories, and more standardised LCA practices to ensure meaningful comparisons and support the sustainable development of biocomposites.

## 1. Introduction

In recent years, research for environmentally sustainable materials has gained significant momentum, driven by the urgent need to mitigate the effects of climate change and reduce dependence on fossil fuels. Among these materials, biopolymer matrix biocomposites have emerged as a promising alternative to traditional composites, offering the potential to reduce environmental impact through the use of renewable resources. More studies are focusing on biocomposites, which consist of natural reinforcing materials and biopolymers, and their application in various industries, including automotive [1], construction [2], and packaging [3]. The advantages of biocomposites, where both parts are biodegradable and bio-based, include biodegradability and raw material availability [4]. However, despite ongoing political calls to replace fossil-based polymers with biological alternatives, it is crucial to determine whether biopolymers and their biocomposites can be produced in a socially acceptable manner, with positive environmental and economic impacts [5].

Over the past decade, research on biocomposites has moved from fundamental investigations of material properties to applied sustainability strategies, reflecting a broader shift in the scientific community toward circular economy principles and life cycle thinking [6]. Between 2012 and 2014, studies primarily focused on understanding the properties and interfacial compatibility of natural fibres such as jute, flax, and hemp with polymer matrices, alongside the development of initial processing techniques. The subsequent period, 2015–2020, signified a shift towards performance optimisation and material diversification. The emergence of “hybrid composites,” which combine natural fibres with synthetic fibres or other fillers to achieve better properties, was a notable development. During this phase, research also expanded to include a wider range of raw materials. Particular emphasis was placed on the utilisation of agricultural residues and biomass-derived waste streams as functional fillers, extending the scope beyond conventional fibre-crop sources. In addition, advanced manufacturing techniques, such as additive manufacturing or 3D printing of biocomposites, became a significant area of research [7].

Since 2020, this field has shifted towards sustainability science. Although material characteristics remain important, keywords such as “sustainability,” “circular economy,” “recycling,” and, in particular, “life cycle assessment” have become widespread. This trajectory represents a shift from simply addressing the issue of material strength to exploring true sustainability and EoL implications. “Biopolymers,” “nanocellulose,” and “biocomposites” are central research topics closely linked to sustainability and environmental impact. This progress highlights that developing biomaterial alone is not enough; it is also important to assess their environmental performance throughout their life cycle, from raw material acquisition to end-of-life management, to ensure true environmental integrity [6,8].

Despite the renewable and biodegradable nature of biocomposites, their environmental benefits are not guaranteed. Factors such as energy-intensive processing, chemical treatments for fibre–matrix adhesion, and agricultural practices can offset potential advantages over conventional materials [9,10]. Consequently, comprehensive LCA has become a critical tool for assessing the environmental impacts of biocomposites, identifying hotspots across their value chain, comparing them to synthetic composites or traditional plastics, and guiding the development of more sustainable production and EoL strategies [8,11].

Based on the EU policy and strategic international regulations, LCA is transitioning from a voluntary methodological choice to a formal regulatory obligation, requiring companies and developers of new materials and products to systematically collect, analyse, and validate life-cycle data. LCA becomes a key decision support tool in the development of biodegradable biocomposites. The EU’s policy context is based on the Bioeconomy Strategy [12], the Clean Industrial Deal [13] and the Product Environmental Footprint (PEF) guidelines [14], all of which promote harmonised life cycle-based environmental impact assessment. Comprehensive LCA of the environmental impact enables the comparison within different biocomposites and with conventional fossil-based biocomposites and plastics, thoroughly assessing critical aspects such as global warming potential, land use changes and end-of-life biodegradability or recycling scenarios [2,15]. Recent research on natural fibres and PLA biocomposites is indispensable for assessing environmental impact hotspots and avoiding burden shifting, in areas such as the construction and energy sectors, where material interchangeability is encouraged by policy measures [16,17,18].

The coordination of scientific research and regulatory measures is becoming extremely relevant in order to supply new materials and products made from them to the EU market, which is focused on a climate-neutral bio-economy. The Ecodesign for Sustainable Products Regulation (ESPR) [19] and its Digital Product Passport (DPP) framework will introduce life cycle-based requirements across priority product groups (electronics, batteries, textiles). Expanding whole-life-cycle rules in national construction legislation will further drive demand for LCA-based environmental product declarations [20,21]. Complementary initiatives—including the Circular Economy Action Plan, the EU Taxonomy, and forthcoming Green Claims rules—reinforce this shift by requiring transparent, verifiable life cycle evidence for compliance and market access [22].

Nevertheless, despite advances in mechanical performance, LCA is still not widely adopted for developed biocomposites, partly due to challenges in data collection, justification of initial assumptions, and proper definition of system boundaries [23]. By identifying methodological challenges and highlighting opportunities for improving environmental assessments, this article fills a key knowledge gap and contributes to the development of biocomposites that are not only mechanically efficient but also truly sustainable within circular economy systems [8,11,24].

This review synthesises current knowledge on LCA application strategies for agro-waste-reinforced biodegradable polymer composites, highlighting current practices, identifying common methodological challenges and opportunities for robust environmental impact evaluation, thereby contributing to the development of sustainable material innovations. The closed cycle of biological materials is set as one of the priorities and opportunities shifting from the linear to circular economy, so there is a need to compare novel materials with each other. The main aim of this article is to reveal the opportunities and challenges associated with LCA analysis of newly developed materials and to determine how existing procedures and databases meet the need for a clear assessment and comparison of the environmental impact of different biocomposites.

In this review, environmental LCA has been chosen as the main environmental assessment strategy due to its widespread adoption and proven ability to provide a comprehensive, cradle-to-grave evaluation of environmental impacts associated with materials. LCA enables quantitative analysis across all life cycle stages, supports hotspot identification, and informs sustainable decision-making and circular economy practices. While other Environmental Assessment Strategies (EASs), such as Strategic Environmental Assessment (SEA), Systematic Sustainability Assessment (SSA), and Safe and Sustainable by Design (SSbD), represent emerging approaches, they are less commonly applied in practice. These methods will be considered in this review only when they appear in combination with LCA in the analysed studies. Consequently, the primary focus of this paper is a critical review of LCA procedures and their application in evaluating material-related environmental impacts.

## 2. Materials and Methods

### 2.1. Scope

This review focuses exclusively on bio-based and biodegradable polymer composites reinforced with agro-waste, as they have real potential for biodegradation at the end of their service life and reduce the environmental impact compared to conventional or partially synthetic composites. In this study, “agro-waste” refers to the leftover materials from the production, processing, and consumption of agricultural products such as fruits, vegetables, grains, meat, dairy, etc. [25].

The assessment of scientific literature in this field was conducted through a bibliometric analysis of scholarly articles. The primary objective of the methodological approach was to collect, analyse, and synthesise existing LCA studies that evaluate the environmental performance of these agro-waste-reinforced biocomposites. A comprehensive literature search was performed through the Scopus online database using the Advanced Search function, targeting peer-reviewed journal articles. The search focused primarily on recent publications from the last five years, in order to capture current research trends and recent advances in LCA of agro-waste-based biocomposites. The search strategy combined three key concept groups: (a) life cycle assessment terminology, (b) composite material type, and (c) natural fibre or agricultural-based reinforcement, including the most popular agro-waste defined in the previous article [26]. The following search string was used: (“life cycle assessment” OR “life-cycle assessment” OR “LCA”) AND (“biocomposite” OR “bio-based composite” OR “bio based composite” OR “bio composite” OR “natural fibre composite” OR “natural fibre composite” OR “green composite”) AND (“agricultural waste” OR “agricultural residue” OR flax OR cellulose OR hemp OR jute OR sisal OR kenaf OR “rice husk” OR bamboo OR coconut OR ramie OR cotton OR banana OR “pineapple leaf” OR sugarcane OR wheat OR “fruit bunch” OR “oil palm” OR “chicken feather”). The search was applied to titles, abstracts, and keywords.

### 2.2. Inclusion and Exclusion Criteria

The initial literature search resulted in a total of 3200 articles. During the first screening stage, all retrieved records were evaluated based on their titles and abstracts to assess their relevance to agro-waste reinforced biodegradable and bio-based composites and their application of LCA methodology. Non-peer-reviewed publications and duplicates were also omitted. In the second screening stage, the full texts of the selected articles were examined in detail. At this stage, studies focusing on non-fully biodegradable or partially bio-based composites, as well as those lacking a clear LCA framework, were excluded. After applying the five-year publication period filter and the defined inclusion and exclusion criteria, 43 articles were retained and listed for detailed qualitative and methodological analysis.

### 2.3. Data Extraction and Categorization

For the analysis of the selected articles, data extraction was performed using a structured synthesis table. The criteria and key guiding questions used for data extraction are presented in Table 1. These criteria covered system boundaries, functional units, data sources and quality, impact categories and assessment methods, assumptions and allocation procedures, sensitivity analysis and integration with broader sustainability metrics.

The information obtained was compiled into a single document to facilitate cross-comparison of studies and identify methodological trends, common assumptions, gaps, and opportunities for developing the application of LCA in biocomposite research. In addition to methodological aspects, the main conclusions and key results of the LCA reported in each study were also analysed to identify dominant trends in environmental impact, frequently mentioned hot spots, and conclusions regarding the sustainability of biocomposites.

## 3. Global Patterns of Agricultural Residues and Localization

**Figure 1:**
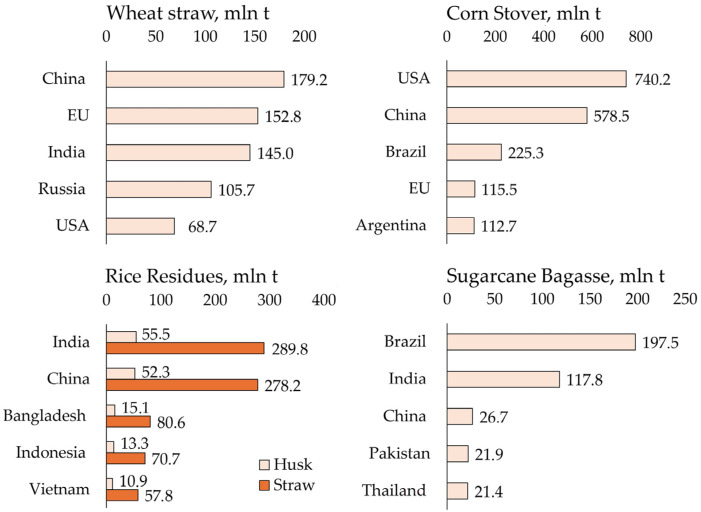
Main agro-wastes and their global production.

Agricultural waste has great potential for the production of biocomposites, as it is abundant and underutilised. Global agricultural waste production is estimated at approximately 2800 million tons per year [27]. Among agricultural wastes, rice straw, wheat straw, corn shavings, and sugarcane bagasse are the dominant global raw materials for biomaterials. Their production is closely linked to regional crop patterns, resulting in pronounced geographical specialisation [28]. The main producers are North and South America, East, Southeast, and South Asia, as well as Eastern Europe [29]. Using FAOSTAT production data for 2024 and the average residue-to-yield ratio applied in the FAO Bioenergy and Food Security Rapid Assessment Tool (BEFS RA), the most common agricultural residues and their main regions of production were calculated and presented in Figure 1.

Asia is the largest global source of rice residues, particularly rice straw and rice husk, due to intensive rice cultivation in countries such as China, India, and Bangladesh. The management of rice straw presents a major environmental challenge in the region, as open-field burning is still widely practiced, leading to severe air pollution and greenhouse gas emissions. At the same time, the large volumes of residuals create significant opportunities for valorisation into biocomposites, biochar, and other bio-based products [30,31]. The high silica content in rice straw is particularly important for its use in materials, as it affects both the reinforcing properties and the processing requirements [32,33].

Cereal residues such as wheat straw are more geographically dispersed but remain concentrated in regions with high wheat production, including China, the European Union, India, Russia, and the United States. China alone contributes approximately 15.73–17.45% of global wheat production [34]. Wheat straw is commonly used for animal bedding, feed, and energy production, and therefore, its use in biocomposite production is limited and necessitates further LCA research.

Corn stover exhibits a similar global distribution pattern, with major production in the Americas (the United States with the Corn Belt region, Brazil, and Argentina) and China. Compared to rice residues, corn stover production is generally more geographically concentrated, facilitating large-scale collection and centralised processing. This spatial concentration can reduce logistical complexity and the associated environmental impact, improving its suitability for large-scale biocomposite production [35]. Nevertheless, transport distances from scattered agricultural fields, seasonal availability, and storage requirements remain important parameters that must be clearly considered in LCA modelling.

Sugarcane bagasse is a highly concentrated agro-industrial residue produced mainly in Brazil, India, China, and Southeast Asia. The bagasse produced during sugar processing is easily collected at processing sites, reducing logistical problems and the associated environmental impact [36].

Agricultural residues can be classified in numerous ways depending on their origin, composition, or intended application in biocomposites. However, from an LCA perspective, a logistics-based classification of agricultural residues provides particularly valuable insights, as it directly influences system boundary definition, inventory assumptions, and impact allocation. Agricultural residues can be categorised into two main groups based on logistics: primary and secondary residuals [37]. The first group includes centralised residues like sugarcane bagasse and rice husk, which are collected during industrial processes and are suitable for large-scale uses such as biorefineries or power plants. The second group consists of distributed field residues like cereal straws and corn stover, which are spread across farmlands. Collecting, baling, storing, and transporting these distributed residues is costly, often leading farmers to use them on-site or burn them in the field. Overcoming these logistical challenges is key to utilising large biomass resources. Therefore, solutions for bagasse can be centralised, while those for straw require decentralised processing, better collection systems, or government solutions to be cost-effective. Given the environmental concerns associated with the disposal of agro-industrial residues, particularly their accumulation and the potential for environmental deterioration, it is highly important to explore their revalorization into sustainable products and bioenergy [38].

From an LCA point of view, this distinction is particularly important because it affects whether cultivation and agricultural management stages are included within the system boundaries. Centralised agro-industrial residues are often treated as by-products or wastes, with cultivation impacts partially or fully allocated to the primary product, whereas distributed field residues may require explicit consideration of additional collection operations and, in some cases, allocation of agricultural burdens. These methodological choices can significantly influence the calculated environmental impacts and the interpretation of sustainability performance.

## 4. Methodological Framework for LCA of Biocomposites

LCA is a tool used to evaluate the impact of a product or material on the environment, human health, and resource consumption. It considers all stages of a product’s life cycle, from raw material extraction through manufacturing, distribution, and use, to final disposal. According to ISO 14040-14044 [39,40], LCA involves the collection and evaluation of the inputs, outputs, and potential environmental impacts of a product system throughout its life cycle [41]. However, while ISO’s definition of LCA focuses on environmental factors, it does not include social or economic factors [42]. LCA can also serve as a valuable tool in the design process, helping to understand and evaluate the technical solutions used in production. By doing so, it can minimise impacts not only from the product itself but also during its use and at the end of its life cycle [43].

LCA consists of several stages. It begins with a goal and scope definition, where the purpose of the LCA is established, and the system boundaries and functional unit are determined [42]. At this stage, it is also important to properly select impact categories and their indicators [42]. Next, during the inventory analysis (LCI), data is collected on all inputs and outputs throughout the life cycle stages, including raw material extraction, manufacturing, transportation, use, and disposal. This is followed by the impact assessment (LCIA), which evaluates the potential environmental impacts associated with the inputs and outputs identified in the inventory analysis. Finally, the interpretation stage involves analysing the results to draw conclusions, make recommendations, and identify opportunities for reducing environmental impacts.

Using the ISO 14040–aligned data extraction framework (Table 1), a systematic analysis was done of recent LCA studies on biocomposites to assess methodology, data completeness, and the transparency of reported results.

### 4.1. Goal and Scope Definition

The first step in performing an LCA of biocomposites or any other material is to clearly define the goal of the analysis. In LCA, the objective influences all subsequent methodological decisions, from defining the system boundaries to selecting data and interpreting results. A literature review identified three types of objectives: comparative assessments aimed at comparing biocomposites with traditional materials or alternative biosystems [44], hotspot identification and process-oriented assessments that focus on specific production stages or technological innovations [45,46] and assessments of the overall sustainability performance of biocomposite products [5]. Many published studies combine more than one goal within a single LCA [47].

**Figure 2:**
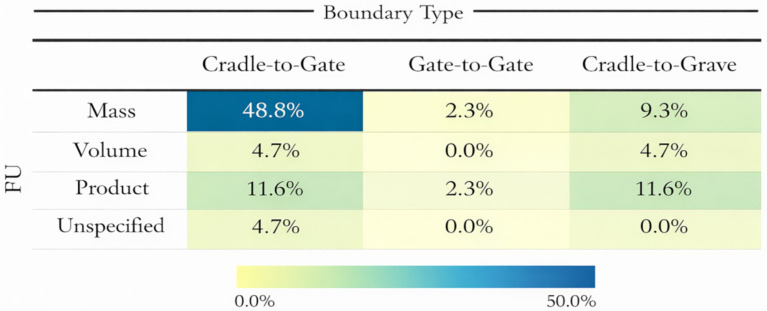
Distribution of functional units and system boundaries.

The next important stage in an LCA is the selection of the functional unit (FU). The FU is a key element of LCA methodology, as it defines the reference against which all material and energy flows are normalised. In studies of biocomposites, the FU may be defined on the basis of mass [5,48], volume [49], or a finished product or component [23]. The distribution of FUs across the articles and their connection with system boundaries are presented in Figure 2. The literature review shows the dominance of mass-based FUs, commonly defined as 1 kg of biocomposite material. Mass-based FUs widespread can be explained by compatibility with existing LCI datasets.

However, several studies favour FUs based on volume or products over mass, as differences in composite density can be significant [44,50]. Nevertheless, FUs based on mass, volume, and simple products may still fail to provide functional equivalence when comparing biocomposites with different mechanical properties, durability, or expected service life. To overcome these limitations, an increasing number of recent studies use performance-based or service-oriented FUs that better reflect the actual function performed by the material or product. For example, Nlandu et al. [45] defines the functional unit based on achieving specific mechanical thresholds (Young’s modulus > 3200 MPa and tensile stress > 52 MPa), while Liu et al. [51] and Kamau-Devers et al. [52] normalise environmental performance according to equivalent flexural stiffness or mechanical property equivalence relative to conventional composites. Application-oriented functional units have also been adopted, including automotive panels delivering equivalent load-bearing capacity [53], ship hulls designed to meet defined operational performance requirements [54], and packaging systems defined by delivered volume or service functionality rather than mass alone [23,43].

The system’s boundaries of the analysis define which processes and impacts will be included in the assessment, such as raw material extraction, manufacturing, transportation, use, and EoL disposal. The most commonly applied system boundary in the literature is cradle-to-gate, which includes raw material extraction and processing, biopolymer production, natural fibre cultivation and treatment, compounding, and composite manufacturing. In a cradle-to-gate LCA, the assessment stops at the exit gate of the product manufacturing company, excluding the downstream phases of distribution, use, and EoL [48]. The selection of the approach depends on the goal of the analysis. If the goal is to analyse new processing technologies or new additives, a cradle-to-gate approach is typically used [9,55]. However, cradle-to-gate boundaries do not consider potential benefits associated with the use phase or EoL of biocomposites, such as weight reduction effects, biodegradability, compostability, or recyclability potential. While this review maps how different system boundaries (from gate-to-gate to cradle-to-grave) are applied rather than prescribing a single approach, we underscore that even lab-scale LCAs of agro-waste-derived biocomposites should include indicative end-of-life scenarios, particularly biodegradability, to identify the limits of degradation, potential EoL benefits, and any additional environmental trade-offs.

To address these limitations, an increasing number of recent studies adopt cradle-to-grave system boundaries, which include the full life cycle of the biocomposite product. This approach is particularly relevant for application-oriented LCA, such as those focused on automotive, packaging, or consumer products, where differences in product life span, maintenance requirements, or EoL pathways can significantly impact overall environmental performance. However, a literature review shows that only about a quarter of studies apply a cradle-to-grave perspective, highlighting that this comprehensive system boundary is still underrepresented in LCA studies of biocomposites.

### 4.2. Life Cycle Inventory

#### 4.2.1. Foreground Process Coverage

Inventory analysis involves the systematic collection and quantitative assessment of all relevant input data (raw materials, energy, and water) and output data (emissions to air, water, and soil, as well as waste streams) associated with each stage of a product’s life cycle. Given that biocomposites consist of at least two different components, namely a polymer matrix and a reinforcing phase, the inventory analysis is often structured accordingly.

**Figure 3:**
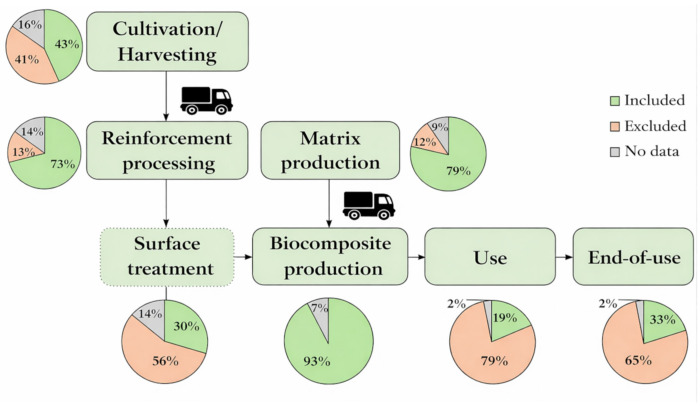
Distribution of system boundaries and main stages across the analysed articles.

For reinforcing materials made from agricultural waste, the inventory typically includes crop cultivation, harvesting, transportation, fibre processing, and, in some cases, surface treatment [42]. For the matrix component, inventory data typically covers polymer production and subsequent processing steps, followed by biocomposite production. When cradle-to-grave system boundaries are applied, the inventory may also include use and EoL stages. Figure 3 presents system boundaries considered in the analysed studies, together with the percentage of articles in which each stage was included, excluded, or not reported.

Crop cultivation can have a significant environmental impact, as it involves activities such as ploughing, sowing, irrigation, fertilisation, and pest control. These processes lead to greenhouse gas (GHG) emissions from fuel combustion and fertiliser application, as well as eutrophication and ecotoxicity impacts due to nutrient runoff and agrochemical use [42,56]. However, the magnitude of these impacts is highly dependent on crop type, geographical location, agricultural practices, and modelling assumptions. For example, La Rosa et al. [57] assumed that fertilisers and pesticides were not required for hemp cultivation, thereby significantly reducing the environmental burden of the cultivation stage.

Almost half of the studies published between 2020 and 2025 included the cultivation step in the LCA [47,58,59]. In contrast, the cultivation step is usually excluded through the application of a zero-burden approach when agricultural residues or agro-industrial by-products are used, such as coffee silverskin [48], durian fruit skin [50], coconut mesocarp [60] or vine shoot particles [23]. Some authors omit this step to reduce uncertainty, as LCI databases often lack representative processes for modelling cultivation [47]. However, recent discussions within circular economy research [61,62] suggest that the validity of the zero-burden assumption is increasingly context-dependent, particularly as certain waste streams acquire economic value, which may require consideration of appropriate allocation methods. That indicates the need to take this aspect into account in the further research of agro-waste as feedstock for biocomposites.

Another important aspect of inventory analysis concerns the processing of reinforcement materials. Especially for agro-waste-derived reinforcements, additional chemical and/or physical treatments are often required to improve compatibility with the polymer matrix. The processing requirements vary significantly depending on plant species, treatment method, and intended application [42]. Washing, grinding, drying, or chemical surface modification can substantially increase energy use and environmental impacts [55,63]. One third of the analysed articles used surface treatment for reinforcements, which can significantly increase the impacts associated with fibre processing. For example, Nlandu et al. [45] included fibre mercerisation in their LCA inventory.

In parallel, the production of the polymer matrix generally includes raw material extraction or biomass processing, transportation, polymer synthesis, and palletisation [42]. These processes often require significant amounts of energy, chemicals, and water, and generate emissions and waste that contribute to impacts on human health and ecosystems [64,65]. Polymer matrix production was marked as a main hotspot in LCA in several articles [23,44,52,66,67]. However, Raue et al. [68] excluded matrix production as the polymers have similar melting temperatures and therefore exhibit similar energy consumption during the manufacturing process.

The compounding and biocomposite manufacturing stages are also frequently identified as environmentally significant. Compounding usually involves extrusion processes to combine the polymer matrix, reinforcement, and additives into pellets, followed by shaping techniques such as injection moulding or compression moulding [69]. These stages are energy-intensive and can contribute substantially to overall impacts due to electricity consumption, material losses, and emissions associated with additives such as plasticizers or compatibilizers [70]. Therefore, almost all analysed articles included this stage and are marked as one of the main hotspots in LCA [48,52]. However, Marinelli et al. [71] assumed that injection moulding is equivalent across the compared systems and therefore can be excluded from the analysis.

The use phase is often simplified or excluded in biocomposite LCAs, particularly when the compared materials are assumed to perform similarly. For example, Salwa et al. [43] excluded the use phase for green biocomposite containers based on Product Category Rules for food-contact plastic containers. In many cases, this stage is limited to transportation impacts or omitted entirely [23,42]. It is clearly seen in Figure 3 that while the end-of-use stage was included in 33% of the articles, the use stage was considered in only 19%. Nevertheless, durability and service life can significantly influence environmental performance, as longer-lasting materials reduce the need for replacement and maintenance. Limited knowledge of long-term performance and degradation behaviour remains a challenge for modelling of the use phase [42].

The EoL stage should reflect realistic disposal and recovery practices for the assessed material. When both the matrix and reinforcement are biodegradable, composting is commonly analysed as a potential EoL option [66]. Beigbeder et al. [72] evaluated four main EoL scenarios—incineration, landfill, composting, and recycling—and concluded that recycling generally offers the lowest environmental impacts. However, due to technical limitations in recycling biocomposites, composting is often considered the most favourable alternative. Incineration with energy recovery is also frequently included, as natural fibres store biogenic carbon during growth, which can be partially recovered as energy at the end of the composite’s service life [42].

During the inventory analysis stage of LCA, it is crucial to develop a flow rate that includes all inputs and outputs. Additionally, potential impact reductions through by-product utilisation should be considered. For example, Ita-Nagy et al. [55] included the recovery of energy and NaOH from black liquor generated during bagasse fibre processing, as well as dissolved hemicellulose, which are valuable for industrial applications.

#### 4.2.2. Data Sources and Software

There are five main categories of data sources for LCA analysis:

1. Literature;
2. Estimations by authors;
3. Laboratory experiments;
4. Databases (e.g., Ecoinvent, Agri-footprint, US Life Cycle Inventory database);
5. Manufacturers.

All these data sources can be classified into primary and secondary sources. Primary data refers to specific information directly collected for the study, such as energy consumption, material usage, or waste generation measured at a specific facility, laboratory, or pilot scale [73,74]. This type of data is particularly valuable for processes directly controlled by the researchers and is typically highly specific and accurate [73]. In the reviewed literature, primary data was most commonly derived from laboratory experiments, pilot-scale trials, and manufacturer or process measurements. Examples include experimental drying processes [45,71,75,76], mechanical performance testing [77,78,79,80], and pilot-scale composite manufacturing trials [68,81]. These datasets often capture detailed processing parameters such as energy demand, processing efficiency, and material conversion rates.

Secondary data, conversely, are sourced from the existing literature, commercial LCA databases, such as Ecoinvent or GaBi, or other generic published datasets [82]. They are typically used to model background processes, where direct measurement is impractical or impossible, such as electricity production, raw material extraction, transportation, and waste management [83].

**Figure 4:**
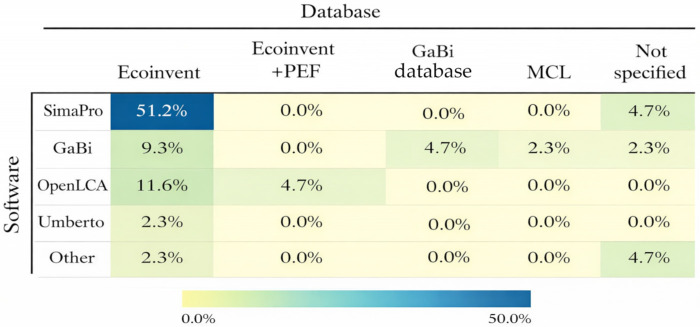
Connection between databases and software.

Across all 43 reviewed articles, secondary LCI data was predominantly sourced from the Ecoinvent database (76.7%). However, the use of databases is closely linked to the choice of LCA software (Figure 4). SimaPro is the most commonly used software and is almost always combined with the Ecoinvent database. Although GaBi software enables the use of its native GaBi database, the review shows that Ecoinvent is more commonly applied, with occasional use of the MCL database as well. OpenLCA was used in 16.3% of the reviewed articles, mainly in combination with Ecoinvent. The main advantages and weaknesses of the most popular software are described in Table A1. Overall, this pattern indicates a strong methodological reliance on Ecoinvent regardless of the software platform selected. This consistency improves comparability across studies but may also limit database diversity and sensitivity to region- or technology-specific background data.

While established databases provide extensive and methodologically rigorous datasets [83], they introduce challenges related to geographical, temporal, and technological representativeness, which can affect the accuracy and comparability of results across studies [84,85]. Therefore, researchers often triangulate data from multiple sources to enhance the validity and reliability of their findings [86,87].

A hybrid data model combines primary and secondary sources. Primary data—such as experimental measurements, pilot-scale processing parameters, and laboratory energy consumption—were used to model foreground biocomposite production processes. These were complemented by secondary datasets from databases, the literature, and supplier inventories to represent upstream and downstream systems, including raw material production, electricity generation, transportation, and end-of-life treatment. This hybrid approach was employed in nearly all reviewed articles (95%) because biocomposite LCAs require both high-specificity foreground data for process accuracy and comprehensive background datasets to ensure system completeness.

The characteristics and reliability of LCA data also depend on the production scale considered. Almost half of the analysed articles (42%) combine laboratory-scale experiments with industrial-scale modelling or secondary industrial datasets. In practice, this means that many studies develop and test materials under controlled laboratory conditions but then use industrial-scale assumptions to estimate environmental impacts. This approach reflects an effort to connect early-stage material development with potential real-world production. However, it also highlights an important methodological issue, as laboratory processes are often less energy-efficient and less integrated than commercial manufacturing systems.

Overall, laboratory-scale research remains dominant within the dataset, as 70% of studies include laboratory experiments either alone or in combination with other scales. In contrast, pilot-scale investigations are less common, appearing in 23% of articles, either as standalone studies or in combination with laboratory and/or industrial modelling.

Regional Differences in Data Sources and Modelling

A strong regional dependency is evident across the reviewed biocomposite LCA studies, influencing both data selection and environmental impact outcomes. While primary data collection practices varied mainly according to study design, the environmental results themselves were highly sensitive to regional background datasets, particularly electricity production systems.

Electricity grids frequently impact results more strongly than the material systems themselves. For example, switching from the Italian low-voltage grid to the Norwegian grid (98.3% renewable) reduced climate change and fossil resource impacts by more than 90% [71]. In Spain, up to 80% of a product’s climate change impact was attributed to electricity use, with solar and wind scenarios significantly lowering the results compared to the national grid mix [54]. Differences in grid composition also explain why the Philippines (52% coal) showed higher impacts in 15 of 18 categories compared to Egypt, where coal represents only 7% of the mix [88]. Similarly, replacing on-site bioenergy with Brazilian grid electricity increased GWP by 12% [55], while energy recovery optimisation in France led to reductions exceeding 50% in some impact categories [44]. Together, these findings show that electricity assumptions alone can shift GWP results by more than 80–90%, highlighting the importance of using geographically representative electricity data in biocomposite LCAs.

Cultivation geography further contributed to regional variability, with examples including hemp in Canada [89], kenaf in Italy [75], coconut fibre in Colombia [60], miscanthus in Germany [50], flax in France [45], and rice straw systems in Europe [66], all shaped by localised agricultural inputs, yields, and processing conditions. Transport routes added further variation—for instance, PLA produced in Thailand and shipped to Italy [90], or U.S.-produced PLA transported to Latvia [58]—contributing substantially to differences in GWP outcomes.

**Figure 5:**
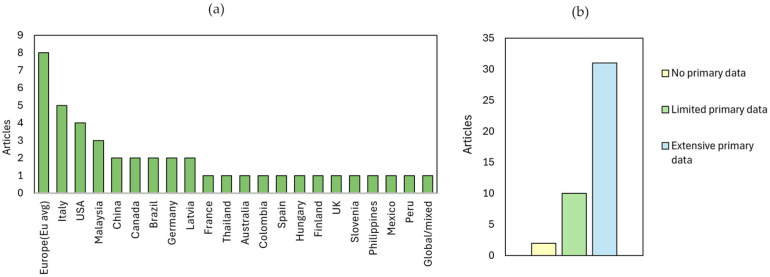
(**a**) regional distribution (**b**) primary data use in LCA articles on biocomposites.

Regions such as Italy, broader Europe, the USA, China, Canada, Germany, and Malaysia appear most frequently in the reviewed literature (Figure 5). This prevalence indicates a reliance on region-specific electricity mixes, localised cultivation and manufacturing datasets, and country-specific transportation assumptions, creating shared “regional fingerprints” across publications. In contrast, regions represented by only one or two articles—such as Peru, Colombia, and Slovenia—are typically associated with niche biomass sources or specialised composite applications.

Primary data intensity also varies regionally, though this is linked more to research capacity than geography itself. Higher levels of primary data collection are more common in studies from Italy [44,71,90], Canada [83], the USA [47,52,91], and Germany [51,92], likely due to stronger research infrastructure, established laboratory and pilot-scale measurement practices, and closer industrial collaboration. Conversely, studies from regions such as Thailand [93], Australia [68], Slovenia [94], and Peru [59] rely more on secondary datasets, often due to limited access to experimental or industrial process data. The intensity of primary data used is presented in Figure 5.

All reviewed articles applied single-region foreground system boundaries. This is consistent with attributional LCA practice, where electricity supply, cultivation systems, and transportation models must remain geographically coherent. Studies using “global” or mixed datasets typically relied on non-country-specific background data rather than modelling fully multi-regional production systems.

#### 4.2.3. Allocation Procedures

Allocation procedures are pivotal in LCAs of biocomposites because these systems are inherently multifunctional—biomass chains and conversion steps yield several products and byproducts—and the way coproducts are handled can meaningfully change results, comparability, and the robustness of conclusions.

Allocation practices across selected papers analysed remain heterogeneous. However, no allocation is still predominant (48.8%). Most no allocation cases are justified by applying a cutoff/zero burden to residues or wastes—inputs are treated as burden-free and upstream impacts are attributed elsewhere [23,59], so agricultural byproducts have “no allocation needed” [43]. Although the zero-burden assumption is widely used when agro-waste is treated as a waste stream, its applicability is increasingly discussed in the context of circular economy systems. As agricultural waste becomes a valuable secondary raw material for bio-based materials and composites, assuming that it carries no upstream environmental burden may oversimplify the real environmental profile of these materials. In such situations, alternative approaches, such as economic allocation or system expansion, may better reflect the environmental trade-offs associated with the use of agro-waste as a resource.

A second common rationale is a single product boundary where no coproducts exist, so allocation is not applicable [78,79,95,96]. Some studies handle multifunctionality via system credits/substitution rather than partitioning, thereby avoiding allocation [81]. Others treat scraps/waste streams as wastes with disposal modelled (no coproduct burden) [46,54,97].

Mass-based allocation is employed in a few papers (13.9%) and appears in two distinct contexts across the reviewed papers. Fractionation is explicitly reported where process streams are split into defined fractions, such as potato pulp and starch [5], or through a general fractionation step [51] with burdens apportioned according to the mass of each fraction. Coproduct formation (composition or multioutput driven) is handled by mass, where the focal product (e.g., short flax fibres) or multiple outputs (e.g., fibre/resin; fertiliser/clamshell) receive burdens proportional to their masses [44,53], with additionally noting system expansion alongside the mass split [88]. These distinctions underscore that mass allocation is used either to partition fractionated streams or to distribute burdens among simultaneously produced coproducts, depending on how the unit process generates outputs.

Economic allocation appears in 11.6% of papers analysed, typically justified by market value [49,53,56,98] or accumulated pricing ratio splits for coproducts [83]. Energy allocation is absent in the analysed papers. Notably, several studies model multifunctionality by credits rather than partitioning [81], while many cases with no allocation would benefit from sensitivity tests (e.g., partial burdens for collection/pretreatment) to strengthen interpretability. Overall, explicit justifications in the rule applying papers tend to be clearer than in those with no allocation, but reporting of data sources (prices, regions, and years) and comparisons to physical alternatives could improve consistency and transparency across the analysed studies.

### 4.3. Impact Assessment

During this stage, the processes discovered through the life cycle inventory study link to various effect categories, which can eventually be connected to environmental impact categories [98]. The LCA process quantifies various inputs and outputs and translates them into a set of potential environmental impacts. Common impact categories relevant to agricultural residue valorisation include GWP, which assesses climate change impact; Eutrophication Potential, which relates to nutrient enrichment of water bodies; Acidification Potential, concerning acid rain; and Fossil Resource Scarcity or Abiotic Depletion Potential [42].

The specific choice of impact categories aligns with the goals of the study and the specific environmental concerns related to biocomposites. This shift underscores the imperative for developing more sustainable material solutions within the composites industry [99,100].

Across the papers analysed, ReCiPe is the most frequently applied characterisation framework, appearing in 39.5% of studies [45,53,55,59,60,94], followed by IPCC GWP in 23.3% [48,60,97], CML [75,90,95] and EF/ILCD (PEF) [46,51,71] each in 18.6%, and TRACI in 11.6% [52,81,83,89,91], CED (energy demand) and IMPACT2002+/World+ appear as complementary in 9.3% [47,48,75,91] and 4.7% [47,91] of papers, respectively.

Method combination reveals a preference for balancing breadth and headline indicators: ReCiPe + IPCC GWP co-occur in 11.6% of papers [5,47,53,55,91], while IPCC GWP + CML [58,77] and IPCC GWP + CED [47,48,91] both appear in 7.0%, and ReCiPe + EF/ILCD (PEF) in 2.3% [78].

In terms of impact category scope, multicategory assessments dominate (69.8%) typically cover acidification, eutrophication (freshwater/marine), human toxicity and ecotoxicity, stratospheric ozone depletion, photochemical ozone and particulate matter formation, land use, water consumption/use, fossil and mineral resource scarcity, and sometimes ionising radiation [51,54,60,68]. Single issue (climate only) studies account for 18.6% [46,66,67,75,79,96,97] focusing on CO_2_eq/GWP as the principal indicator. These shares indicate that authors commonly select ReCiPe/CML/EF for broad midpoint coverage and then add IPCC GWP (and occasionally CED) to foreground carbon/energy KPIs, with EF/ILCD (PEF) used for EU comparability and TRACI where a US-centric data/software context applies [66].

LCA characterisation methods differ in their modelling choices, geographic scope, and coverage of impact pathways (e.g., midpoint vs. endpoint, fate/exposure assumptions, normalisation and weighting). As a result, the selection and increasingly, the combination of methods in LCA studies is used to triangulate findings, test robustness to methodological uncertainty, and tailor results to specific policy or regional contexts.

The analyses of papers synthesise how recent LCA publications deploy characterisation methods and their combinations, the rationale behind these choices, and the implications for result comparability, hotspot identification, and decision support. Overall, ReCiPe [43,47,94] and CML [54,58,77] are frequently applied as standalone, comprehensive methods, reflecting their broad midpoint impact coverage. In contrast, more selective approaches such as IPCC GWP100 [47,48,60] and CED [47,48] often appear in combination to add climate or energy specificity.

Table 2 presents the relationship between different LCIA methods and the impact categories that they cover. Based on the analyses and tables above, the most often evaluated impact categories are climate change, eutrophication (divided into: unspecified, freshwater and marine), acidification, ecotoxicity (general, terrestrial and freshwater) and human toxicity. While cumulative energy demand is used only in few studies as an additional impact category evaluated by the CED method [47,48,60].

**Figure 6:**
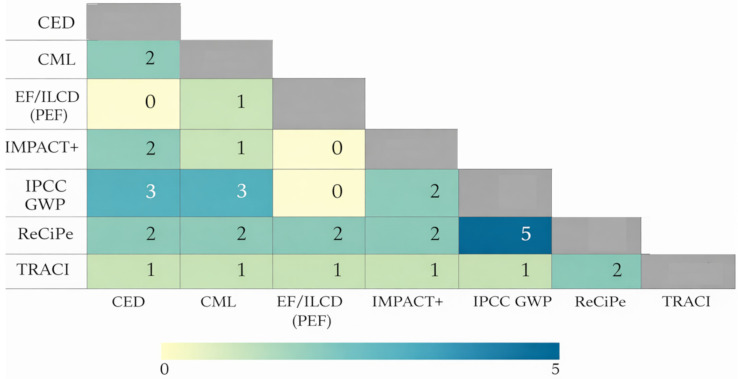
A combination of different LCIA methods.

Figure 6 shows the co-occurrence patterns of LCIA methods across the reviewed studies. The strongest pairing observed is between ReCiPe and IPCC GWP100 [47,56,61], indicating that climate change results are often complemented or cross-validated alongside broader midpoint assessments. Moderate co-occurrence is also visible between CML and IPCC GWP100 [59], as well as between ReCiPe and other midpoint frameworks. In contrast, EF/ILCD (PEF) [51] paired with CML, suggesting more region-specific or policy-driven application contexts.

### 4.4. Interpretation

#### 4.4.1. Uncertainty and Sensitivity Analyses

In the LCA studies reviewed, the treatment of uncertainty and sensitivity analysis is highly heterogeneous. While several studies assess key modelling assumptions, such as electricity consumption, transportation distance, processing energy, material composition, and impact assessment methods, many exclude formal evaluation.

Cross-study comparison reveals consistent patterns regarding the parameters that most strongly influence LCA outcomes. Energy-related variables clearly emerge as the dominant drivers of variability. In particular, the electricity mix and processing energy demand, especially for fibre drying, extrusion, and compounding, are repeatedly identified as the most influential factors shaping environmental performance [54,83]. Quantitative uncertainty analyses using Monte Carlo simulation, pedigree matrices, or variance contribution methods further confirm the central role of energy use, in some cases explaining more than 90% of total variance across impact categories [52,55,92]. In addition, specific process stages, such as solvent combustion or ethanol use in lignin extraction, have been shown to introduce substantial variability, particularly in toxicity-related categories [83].

Transportation distance represents another important parameter, particularly for low-density agro-residue feedstocks, where logistics burdens become proportionally significant [45,58,88]. Similarly, agricultural inputs, including fertilisation practices and biomass cultivation conditions, contribute notably to eutrophication and acidification outcomes. Fibre pre-treatment and chemical modification processes, such as the use of compatibilisers, alkali treatments, and surface modifications, also influence toxicity-related impact categories, although these parameters are assessed less consistently across studies [45,59,83,96].

In contrast, parameters such as minor additives, auxiliary materials, or packaging stages generally show lower sensitivity, unless used in high concentrations or energy-intensive formulations. Overall, although sensitivity analysis is increasingly recognised as essential, its application remains limited, which reduces the comparability and reliability of reported environmental performance. Future research should therefore prioritise systematic sensitivity testing of electricity sources, processing energy intensity, transport logistics, and chemical treatment systems, as these variables most consistently determine result stability.

Methodological limitations, as well as data availability and quality issues, are also widely discussed. Several studies report reliance on secondary datasets or proxy materials, for example, using flax as a substitute for hemp, or generic polymer datasets due to missing inventories, and identify database dependence as a key source of uncertainty [53,101]. Furthermore, variability in natural fibre properties, agricultural practices, and regional energy mixes can significantly influence results but is difficult to capture using average datasets [51,95]. Despite widespread acknowledgement of these limitations, only a minority of studies quantitatively assess how they affect LCA outcomes. A more detailed discussion of these limitations is provided in Section 6.

#### 4.4.2. Integration with Broader Sustainability Metrics

Most analysed publications remain focused only on environmental LCAs, with approximately 9.3% integrating economic considerations [23,53,58,93] and about 2.3% explicitly coupling economic and social dimensions within an LCSA framework [23]. No studies were identified that integrate social dimensions alone. Regarding circularity and EoL, about 42% of publications positively mention CE/EoL in some form [45,47,92,94], yet only ~14% model EoL quantitatively [23,58,80]. Approximately 9% leave the level of EoL treatment unspecified, while around 5% are addressed qualitatively [78,80]. The shares of integration of various sustainability metrics indicate a field still anchored in environmental LCA, with limited but growing attention to economic/social pillars and a clear opportunity to standardise robust CE/EoL scenario modelling across studies.

## 5. Insights from Published LCA Studies on Biocomposites and Environmental Impacts

Nowadays, despite the rapid development and modification of new biocomposites, the analysis of their environmental performance remains limited. As mentioned in the introduction and goal and scope chapter, LCAs of biocomposites used for various purposes usually compare their environmental performance with (a) fossil-based plastics or synthetic fibre composites [23,58,67,83]; (b) neat bioplastics [5,44]; (c) biocomposites produced using alternative processes, additives, or reinforcement contents to identify environmentally preferable formulations [47,50,71,96,102]; or (d) focus exclusively on hotspot identification without a direct comparative reference [43].

### 5.1. Comparison with Fossil-Based Plastics or Composites

Most reviewed studies (60%) compared biocomposites to fossil-based alternatives or traditional plastics. The results generally indicate that natural fillers have better environmental performance than glass fibres, primarily due to the weight reduction in the composites and their low energy demand for production [23,103]. A large share of articles report that biocomposites generally outperform fossil-based composites in terms of GWP and fossil resource depletion [55,58,81,93]. For example, jute-fibre-reinforced polymers exhibited up to 88% lower carbon footprints compared to synthetic alternatives [93], while flax/PLA and hemp/PLA composites showed GWP values of 1.19–1.7 kg CO_2_-eq/kg, compared to 9.14 kg CO_2_-eq/kg for PA66/GF [58].

The beneficial climate performance of biocomposites is often linked to biogenic carbon sequestration during biomass growth and the use of agricultural residues. Hemp-based biocomposites benefited from carbon uptake during cultivation, resulting in approximately 20% improvement in GWP compared to PET-based scenarios [83], while optimised fertilisation strategies further enabled substantial reductions in human toxicity and ecosystem-related impacts [89]. Agro-waste reinforcements such as vine shoots, rice straw, starch, and sugarcane bagasse showed particularly low GWP, as they require minimal processing and avoid burdens associated with dedicated crop cultivation [23,66,95]. For instance, vine-shoot-filled biocomposites reduced GWP by ~25% for PHBV-based trays, ~20% for PLA-based trays, and ~8–9% for PP-based trays at 30 vol% filler content [23].

Beyond climate-related indicators, agro-waste-reinforced biocomposites provide broader environmental benefits, including improvements in human health and resource depletion categories. Fully bio-based composites reduced human health impacts by more than 50% compared to petroleum-based systems, largely due to lower emissions of volatile organic compounds and reduced dependence on non-renewable resources [104]. Comparable reductions were observed for abiotic depletion of fossil resources across multiple product systems [55]. Furthermore, the energy required for producing natural fibres is significantly lower than that for synthetic reinforcements such as glass fibres, reinforcing the environmental advantages of agro-waste-based composites [1,95].

The use of agricultural waste as a reinforcing agent also supports waste valorisation and circular economy principles by transforming underutilised by-products into value-added materials, thereby reducing the burden on waste management and increasing resource efficiency [6,26]. In addition, many composites made from agricultural waste are biodegradable or compostable, allowing for environmentally friendly disposal options and reducing the burden on landfills. When recycled, biocomposites often have the lowest overall environmental impact among the available disposal scenarios [72].

However, despite these advantages, biocomposites do not universally outperform fossil-based materials across all impact categories. Although climate change mitigation is frequently highlighted as a key advantage of biocomposites, reliance on GWP alone may mask other important categories. Several reviewed studies report increased impacts in categories such as eutrophication and acidification [58,83]. The production of biocomposites, particularly those derived from agricultural sources like hemp, involves significant nutrient runoff due to the use of nitrogen fertilisers. This runoff can lead to eutrophication in nearby water bodies, impacting aquatic ecosystems [89]. Additional contributions arise from chemical treatments and wastewater emissions.

The primary contributor to acidification in biocomposite production is the energy consumed during processing, which leads to CO_2_ emissions [50]. For example, cellulose acetate–Miscanthus composites exhibited higher impacts than polypropylene–glass fibre composites across all assessed categories, with cellulose acetate production identified as the dominant hotspot due to acetic anhydride synthesis [51]. Likewise, laboratory-scale production and energy-intensive synthesis routes can offset the environmental benefits of bio-based systems, as shown for AgNP-containing biocomposites where nanoparticle synthesis dominated overall impacts [68]. Enhancements in material composition, such as the addition of epoxidised palm oil, can reduce the energy required for production, thereby lowering the acidification potential [50].

This burden-shifting effect underscores the importance of multi-impact assessment frameworks. A balanced sustainability evaluation, therefore, requires consideration of both climate and non-climate indicators to avoid problem shifting between environmental domains.

### 5.2. Comparison with Bioplastics

When compared to neat bioplastics such as PLA, PHBV, or PBS, biocomposites frequently show reduced environmental impacts, particularly in climate change and fossil resource scarcity, due to the partial replacement of energy-intensive biopolymer matrices with natural reinforcements [23,44,47,48,90]. These benefits are most pronounced when low-burden, waste-derived, or residue-based reinforcements are used [23,44].

Several studies report that incorporating natural reinforcements into PLA reduces GWP by approximately 7–25%, due to the high upstream impacts of PLA production [23,44,47,90]. For example, hemp straw–PLA composites exhibited lower impacts across most ReCiPe 2016 categories compared to pure PLA, with PLA production identified as the primary hotspot [47]. Similarly, increasing biofiller content in SCG/PBS systems reduced cumulative energy demand and carbon footprint by 7–8% [48].

However, when natural reinforcements are added to neat biopolymer matrices, results vary considerably and are highly dependent on system boundaries, allocation approaches, and processing requirements. Chen et al. [5] showed that valorised potato pulp used as a filler in PLA and PHA biocomposites can increase overall greenhouse gas emissions due to the high consumption of thermal energy and electricity required to dry the pulp, offsetting the benefits of reducing biopolymer content. This finding illustrates that additional processing steps associated with natural reinforcing materials can negate the expected environmental benefits. David et al. [23] also showed that incorporation of vine shoots became environmentally beneficial only beyond certain filler contents, namely around 5.5% for PLA and PHBV and 20% for PP in terms of global warming potential.

### 5.3. Comparison Between Biocomposites

Comparisons among biocomposites are commonly conducted to identify the most environmentally favourable option among alternative bio-based material systems, rather than to benchmark against fossil-based references. These studies typically focus on evaluating the influence of fibre type, fibre content, surface treatment, and processing route on environmental performance [45,50,71,96,102].

Several studies demonstrate that processing choices play a dominant role in determining environmental impacts. In particular, chemical surface treatments and fibre modifications substantially increase environmental impact due to additional energy consumption and chemical inputs [45,96]. For example, laboratory-scale surface treatments were shown to significantly increase impacts, whereas optimising drying and solvent recovery stages led to notable reductions [45]. The use of additives can also impact, for example, the incorporation of epoxidized palm oil in PLA/durian skin fibre biocomposites, which reduced environmental impacts by lowering electricity demand during fabrication [50].

Many studies identified energy use during manufacturing as a key hotspot in intra-biocomposite comparisons. As an example, cold blending combined with a high share of renewable electricity can reduce environmental impacts by up to 90% compared to conventional melt blending [71]. This highlights that energy sourcing can outweigh material selection in determining overall environmental performance.

Material formulation also plays a critical role. Increasing the reinforcement content generally reduces impacts by lowering polymer demand. This trend was confirmed by Ingrao et al. [48], who showed that increasing spent coffee grounds content (20–60 wt%) in PBS-based biocomposites reduced both carbon footprint and cumulative energy demand, despite electricity for drying and injection moulding remaining a key hotspot. Among starch-based systems, corn starch combined with optimised processing routes demonstrated the highest eco-efficiency, while the inclusion of agave fibres further improved environmental performance [102].

Table 3 presents representative LCA examples for each type of biocomposite comparison, illustrating the environmental performance, key hotspots, and methodological approaches.

## 6. Challenges and Limitations in Conducting LCAs for Biocomposites

Literature analysis has revealed a significant number of challenges and limitations in conducting LCA for biocomposites, as listed below.

1. Incomplete system boundaries and uncertainty in EoL modelling

Many studies apply cradle-to-gate system boundaries or focus on manufacturing, excluding use, distribution, and EoL stages, which limits the completeness of their conclusions. This is particularly problematic for biocomposites, where biodegradability and waste management are among the key benefits for sustainable development.

The EoL stage remains one of the most important but often overlooked components in the LCA of biodegradable and compostable materials [52,55,88]. For biocomposites, accurate EoL modelling is important for determining their true environmental performance compared to fossil fuel-based materials [55]. However, due to the lack of reliable and standardised data on biodegradability, recycling behaviour, and waste treatment pathways, many studies exclude this stage or apply simplified assumptions, limiting the completeness and policy relevance of their conclusions.

Even when EoL is considered, it is frequently modelled using simplified or idealised scenarios. In many cases, complete biodegradation under controlled industrial composting conditions is assumed [23]. In practice, biodegradation is a complex and context-dependent process. Degradation rates vary significantly depending on polymer chemistry, crystallinity, composite structure, fibre content, environmental conditions (industrial composting, home composting, soil, landfill, or aquatic environments), temperature, moisture, and microbial activity [105,106]. For example, PLA typically requires well-managed industrial composting conditions for efficient degradation, while PBAT does not degrade in anaerobic digestion [105]. Similarly, the presence of additives, compatibilisers, or surface treatments may influence degradation pathways but is rarely accounted for in LCA modelling [107,108,109]. Future studies should integrate experimentally validated biodegradation data and scenario-based waste treatment modelling (industrial composting, landfill with/without gas capture, mechanical recycling) to reduce uncertainty and enhance the policy relevance of biocomposite LCAs.

2. Incomplete accounting of plastic waste impacts

One of the most important issues when comparing biocomposites and conventional plastics is the incomplete consideration of the environmental impact of plastic waste [52]. Many studies assume that all plastic waste is properly managed, without taking into account actual littering and release into terrestrial and aquatic ecosystems [23,110]. The long-term effects of plastic pollution, particularly the formation and accumulation of microplastics, are not typically integrated into standard impact categories [23,110].

To date, there is no officially standardised LCA methodology for assessing the potential impacts of plastic emissions on the environment [111]. Over the past few years, several studies have focused on incorporating the impacts of microplastics and plastic litter into LCA by developing polymer-specific characterisation factors (CFs). These CFs are calculated for various environmental compartments and regions, taking into account factors such as polymer type, particle size, and shape [111,112].

Schwarz et al. [113] developed CFs for major microplastics, including PP, LDPE and PET, and implemented them within the ReCiPe 2016 method. Their approach combines the Simplebox4Plastics multimedia fate model with species sensitivity distributions to assess environmental impacts across different environmental compartments. These models account for the dynamic degradation and fragmentation of microplastics, processes that strongly influence their environmental persistence and impacts [111,114].

Additional efforts are being advanced through international methodological initiatives such as the Marine Impacts in Life Cycle Assessment (MarILCA) working group. This initiative aims to systematically integrate plastic litter into LCA, with a primary focus on marine ecosystems [115,116]. Beyond chemical ecotoxicity, MarILCA expands the scope of impact assessment by including a category addressing the physical effects of plastics on biota via external pathways (entanglement, smothering) and internal pathways (ingestion) [116]. According to the MarILCA methodology, CFs should include three components—fate, exposure, and effect factors. For example, Corella-Puertas et al. [111] provide CFs for physical effects covering eleven polymers, three particle shapes, and five size classes, while Pellengahr et al. [117] determined fate factors for three biodegradable polymers.

Some studies have also introduced screening-level characterisation factors to facilitate the early inclusion of plastic litter impacts in LCA. Salieri et al. [118] proposed a freshwater ecotoxicity CF for microplastics and applied it in the life cycle assessment of a polyester T-shirt.

Case studies show that including plastic litter impacts in LCA can significantly alter assessment outcomes, highlighting the importance of detailed modelling [112,119]. However, substantial uncertainties remain, particularly regarding emission inventories, fragmentation processes, particle heterogeneity, and long-term environmental behaviour, indicating the need for further methodological refinement.

In addition, LCA does not typically consider emissions from the degradation of petrochemical polymers in the environment, despite evidence that plastics release methane and ethylene when exposed to sunlight in both air and water [23,59]. This leads to an underestimation of climate-related impacts. As the bioplastics can be recycled, composted, and incinerated. Therefore, the modelling and explicit evaluation of various end-of –life strategies could better lead to the science-based decision making how to manage the impacts of the biocomposites after the initial usage.

3. The need for balance between mechanical performance and environmental impact

The development of biocomposites is often limited by the need to balance mechanical properties and environmental sustainability. The use of additives, including coupling agents, compatibilisers, plasticisers, and surface modifiers, is often required to enhance fibre–matrix adhesion, mechanical strength, and processability. However, many of them are fossil-derived and involve energy- and chemical-intensive synthesis routes [43,59]. Their incorporation can increase impacts in categories such as human toxicity, ecotoxicity, and acidification due to upstream chemical production, solvent use and emissions associated with additive manufacturing [120,121]. On the other side, plasticisers can reduce acidification due to improved workability and reduced energy requirements [122,123]. The development of bio-based plasticisers, such as those derived from tartaric acid or waste cooking oil [124,125], can further reduce environmental impacts; however, this aspect should be examined more comprehensively in future studies.

Despite their functional importance, additive inventories are frequently aggregated within polymer datasets or insufficiently reported, limiting transparency in LCA modelling. Future studies could improve accuracy by accounting for additives separately, which would help better capture formulation-level environmental impacts and support more reliable sustainability comparisons [43].

In addition, normalising environmental impact based on a single functional property, such as tensile strength, may mask other relevant performance indicators, including elongation at break and impact resistance, thereby introducing methodological bias into comparative assessments [59].

Polymer-specific characteristics such as molecular weight distribution, crystallinity degree, copolymer structure, and additive formulation can significantly influence processing temperatures, mechanical durability, and degradation behaviour. These parameters indirectly affect energy demand, service life assumptions, and end-of-life modelling in LCA. However, such physicochemical characteristics are inconsistently reported in the reviewed studies, limiting the systematic integration of polymer science variables into life cycle assessments. To improve the reliability and comparability of future inventories, it would therefore be beneficial for studies assessing biodegradable and bio-based biocomposites to report a minimum polymer characterisation set. Key parameters include molecular weight distribution, which influences processability and mechanical performance; glass transition temperature and melting temperature, which determine processing/conditions and thermal stability; and degree of crystallinity, which affects stiffness, ba/rrier properties, and degradation behaviour [126,127,128]. In addition, reporting basic mechanical properties relevant to the functional unit (e.g., tensile strength or Young’s modulus) would support more meaningful functional comparisons between materials. Information on polymer composition, such as copolymer structure, blends, and additive or plasticiser content, is also important because these factors can alter processing requirements, durability, and environmental performance. Providing this basic characterisation information would improve transparency, enable more effective modelling assumptions, and strengthen the integration between material science and environmental assessment of biocomposite systems.

4. Limited primary data and dataset representativeness

A common limitation of LCA for biocomposites is the lack of primary data, as most studies rely on values obtained from literature, general databases, and assumptions rather than direct measurements of material flows, energy, and emissions [43,46,53,68]. This reliance on secondary data reduces the accuracy, transparency, and credibility of environmental impact results, especially for new biopolymers, natural fibres, and chemical compatibilizers [43,68,81]. Direct collaboration with industrial partners, in situ data collection and inclusion of all steps are widely recommended to improve the accuracy and representativeness of LCI [46,53].

The substitution of material-specific datasets with proxy and generic datasets represents another source of uncertainty in biocomposite LCAs [46,51,68]. Examples include replacing hemp-based materials with starch-PLA blends, using generic organic chemical datasets for bio-based resins, or substituting alternative chemicals for monomers and compatibilizers in LCA databases [46,68]. Such approximations can result in over- or underestimation of climate, toxicity, and energy-related impacts, particularly in health-related impact categories [43,46].

5. Uncertainties and sensitivity analysis

Uncertainty in input data has been shown to significantly influence key impact categories in biocomposite LCAs, as demonstrated by Monte Carlo and sensitivity analyses [83]. Without explicit uncertainty management, early-stage LCA models may misrepresent the environmental performance of biocomposite supply chains, undermining their usefulness for decision-making and policy development [43,83]. Sensitivity analysis is crucial for understanding how these uncertainties affect the results. However, only a small number of articles currently include sensitivity analysis, although this trend is increasing over the years. PEF methodology partially helps to harmonise LCA increasing the possibility to make comparisons between different cases using Product Category Rules and guidance.

6. Laboratory-scale data

Many existing LCAs are based on laboratory- or pilot-scale production data, where parameters such as energy consumption and material conversion efficiencies are directly measured [46,51,59,60]. While this improves process specificity, laboratory conditions often rely on simplified processing flows and lack the integration efficiencies typical of large industrial facilities. As a result, LCA outcomes may differ significantly from those observed in optimised commercial production systems optimise [59].

In addition, conventional plastics are produced within highly optimised, mature industrial infrastructures. In contrast, many bioplastics and biocomposites are still manufactured in smaller volumes using less advanced technologies. This difference in technological maturity complicates direct comparisons and may either overestimate or underestimate the real environmental competitiveness of emerging bio-based systems [23]. Consequently, future LCAs should incorporate scale-up scenarios, industrial benchmarking, and prospective modelling approaches to better reflect realistic market deployment conditions.

7. Geographic limitations

Accounting for geographical differences in inventory data and impact modelling is also a significant challenge. Ignoring spatial variations can lead to misleading results, especially for impact categories such as acidification and eutrophication, which are sensitive to local environmental conditions [129,130].

Strong regional variability in biomass cultivation, including differences in climate, agricultural practices, and yields, directly affects emission profiles and input resources [51,59]. Many assessments also rely on secondary or proxy datasets due to limited access to region-specific primary data, particularly for upstream processes such as fertiliser and pesticide use, as well as early-stage material production, which increases the uncertainty of results [46,68,90]. A geographical disconnect can therefore occur between the origin of biomass feedstocks and the background datasets used in LCA modelling. For example, many LCA studies of biocomposites based on agro-wastes from tropical or subtropical regions rely on European or global-average inventory datasets, particularly for electricity production, transport infrastructure, and material processing. This often reflects the limited availability of local experimental or industrial data. Such mismatches may distort impact estimates and introduce additional uncertainty. Expanding the use of region-specific inventory data and regionalised impact assessment methods would significantly improve the representativeness of biocomposite LCAs.

However, there is another side to this argument: studies conducted in a single geographical context may not be generalizable, as biocomposite production may have significantly different impacts when implemented in other regions or in future scenarios of large-scale deployment [95].

8. Difficulties in results comparison across different studies

There is a lack of standardised methodologies for conducting LCA on biocomposites. Variability in the stages included databases, different impact categories, assessment methods, and software makes it difficult to compare studies across different studies.

Because of the lack of harmonised impact categories, some studies use midpoint indicators (e.g., CML), others use endpoint methods (e.g., ReCiPe), and some use IPCC-only climate metrics. ISO standards lack specificity for biocomposites, especially in biogenic carbon accounting and co-product allocation. Standards do not fully address circularity, dynamic system boundaries, or consequential LCA. Biocomposites’ apparent climate gains arise from biogenic carbon uptake and substitution, but the storage is temporary: composting or energy recovery re-emits carbon on near-term horizons. Converting agro-waste into products with defined service lives can delay post-harvest release and extend carbon use; however, without harmonised, time-explicit LCA for biogenic carbon, co-product allocation, and dynamic boundaries, transient buffering is often mistaken for durable mitigation.

9. Functional unit definition and representativeness

One limitation in the LCA of biocomposites is the definition of the functional unit, which is often simplified to indicators based on mass or volume (e.g., per kilogram or per cubic metre of material). More and more studies use the product as the FU, but this does not necessarily reflect the actual service or performance provided by the biocomposite in its intended application, especially when mechanical, thermal, or durability requirements differ significantly between biological and fossil alternatives. Performance-based or service-oriented FUs, which better reflect the functional equivalence of materials at the application level, may be a solution to this problem [53]. These approaches improve functional comparability by linking environmental impacts to the mechanical service delivered, rather than simply to material mass. However, considerable methodological differences remain across studies. Performance indicators, durability assumptions, and service-life definitions often vary substantially, making direct comparisons challenging. Developing harmonised guidelines for performance-based functional units by incorporating mechanical performance metrics, lifetime equivalence, and application-specific benchmarks should therefore be considered as a key priority to enhance cross-study comparability in biocomposite LCAs.

All of the identified challenges pertain primarily to scientific LCA. Nonetheless, they may also affect the applicability of results to the EU market and compliance with regulatory requirements. Although the PEF and DPP frameworks are being developed to streamline implementation, this simplification may compromise the completeness of LCA studies and the robustness of comparative analyses across materials. Conversely, digital transparency mechanisms, particularly the DPP, have the potential to strengthen the inventory phase [131] and support the development of more comprehensive databases, thereby enhancing openness and grounding LCA evaluations in larger volumes of real-market data [132]. Given that this study has focused mainly on methodological aspects of LCA for biocomposites, alignment between EU legal requirements and LCA practice constitutes a relevant topic for future research.

## 7. Conclusions

LCA is an important but methodologically sensitive tool for assessing environmental performance. Although many studies report potential advantages of biocomposites, the results remain highly dependent on methodological choices, data quality, and system boundaries. Studies generally pursue three aims: comparison with alternative systems, identification of process hotspots, and evaluation of overall product sustainability.

Although mass-based FUs dominate due to database compatibility, they fail to reflect the actual performance of biocomposites in real-world applications. This limitation highlights the importance of using performance-based or service-oriented FUs for more meaningful comparisons. Most studies use cradle-to-gate boundaries, identifying hot spots such as polymer matrix production, fibre drying and surface treatment, and energy-intensive compounding. However, cradle-to-grave modelling remains limited, despite its strong impact on climate and fossil resource depletion outcomes through recycling and composting scenarios.

Assumptions regarding inventory remain a major source of uncertainty. One key issue is the inclusion/exclusion of cultivation, which significantly complicates comparisons between different articles. Reliance on laboratory-scale data and proxy datasets further limits representativeness, and formal uncertainty and sensitivity analysis remains rare. Impact assessment is predominantly carried out using ReCiPe, IPCC GWP, CML and EF/ILCD, typically applied through SimaPro and openLCA, and focuses primarily on climate change, eutrophication, acidification, human toxicity and fossil resource depletion.

In terms of results, biocomposites consistently achieve significantly lower GWP and fossil resource use than fossil fuel-based systems. However, there is a higher impact on eutrophication, land use and water, largely related to agricultural inputs, energy-intensive processing, and fibre drying. Compared to pure bioplastics, agro-waste reinforcements typically reduce the carbon footprint and cumulative energy consumption, unless additional drying or chemical treatment offsets these benefits.

Key issues on LCA of biocomposites identified include heavy reliance on laboratory data and secondary inventory data, a predominance of functional units based on mass or volume, limited geographical representativeness of agricultural and energy resources, and frequent exclusion or simplification of use and disposal stages. These limitations constrain the comparability and transferability of results across studies and regions.

Future research should prioritise the development of transparent, region-specific, and industry-scale life cycle datasets, alongside standardised system boundary definitions and improved uncertainty analysis. The integration of EoL scenarios will further enhance the reliability of LCA results. Overall, further methodological harmonisation and data quality are crucial to ensure that LCA can effectively support the sustainable design, implementation and evaluation of biocomposite materials policies.

## Figures and Tables

**Table 1:** Data extraction categories.

ISO LCA Steps	Criterion	Key Questions	Notes for Analysis
Goal and Scope Definition	Goal and Scope Definition	Is the study’s aim clearly stated? What is the functional unit? What system boundaries (cradle-to-gate, cradle-to-grave, etc.) are defined? Is the study conceptual, lab-scale, or market-oriented? Are biocomposites compared with conventional materials using functional equivalence?	Assess clarity and consistency across studies.
Life Cycle Inventory	Foreground Process Coverage	Which life cycle processes are included (biomass cultivation, fibre extraction, compounding, manufacturing, use, EoL)? Are any stages excluded?	Identify gaps in the modelled system and inventory completeness.
	Data Sources and Software	What type of data is used (primary vs. secondary)? Are assumptions transparent? Are datasets region-specific or global? Which databases and software are used?	Identify strengths and gaps in data quality and reliability. Note geographic applicability and representativeness.
	Allocation Procedures	How are co-products, recycling, or multifunctional processes handled? Are allocation rules (mass, energy, economic) justified?	Highlight consistency or divergence in allocation strategies.
Life Cycle Impact Assessment	Impact Assessment Methods	Which impact categories are included (e.g., GWP, eutrophication, toxicity)? Which characterisation models are applied (ReCiPe, CML, TRACI, EcoIndicators)?	Compare methodological choices and coverage.
Interpretation	Uncertainty and Sensitivity Analysis	Does the study include uncertainty or sensitivity analysis? Are methodological limitations acknowledged? Are the conclusions consistent with the goal and scope?	Assess the transparency and robustness of conclusions.
	Integration with Broader Sustainability Metrics	Does the study integrate LCA with economic or social assessments? Are circular economy or EoL scenarios considered?	Identify holistic approaches vs. purely environmental focus.

**Table 2:** Relationship between LCIA methods and impact categories.

	ReCiPe	IPCC	CED	CML	TRACI	PEF/ EF	IMPACT 2002+	IMPACT World+	Hybrid LCA/IO	USEtox	ILCD Midpoint+	SUM
Climate change (GWP)	14	7	5	8	5	6	1	1	2	1	0	50
Eutrophication (unspecified)	12	5	3	6	5	4	1	1	1	0	0	38
Acidification	10	4	4	7	5	3	1	1	1	1	0	37
Ecotoxicity (general)	10	3	4	6	5	1	1	1	1	1	1	34
Human toxicity	11	3	4	5	1	2	1	1	1	1	1	31
Stratospheric ozone depletion	9	4	3	4	5	2	1	1	1	0	0	30
Land use/ Transformation	12	3	3	0	1	2	1	1	1	1	0	25
Photochemical ozone formation/Smog	6	4	2	4	5	1	1	1	1	0	0	25
Resource depletion	10	3	3	2	1	1	1	1	1	1	0	24
Water use/Scarcity	10	3	3	0	1	2	1	1	0	1	0	22
Resource depletion (minerals/metals)	6	2	2	4	1	2	1	1	0	0	1	20
Particulate matter formation	8	2	2	0	1	2	1	1	1	0	0	18
Ecotoxicity (freshwater)	6	2	2	1	1	1	1	1	1	0	1	17
Ecotoxicity (terrestrial)	5	2	3	3	1	0	1	1	1	0	0	17
Eutrophication (freshwater)	6	3	2	0	1	2	1	1	0	0	0	16
Ecotoxicity (marine)	5	2	2	2	1	0	1	1	1	0	0	15
Eutrophication (marine)	6	2	2	0	1	0	1	1	1	0	0	14
Ionising radiation	5	2	2	0	1	1	1	1	1	0	0	14
Cumulative Energy Demand	2	2	2	0	0	0	0	0	0	1	0	7
SUM	153	58	53	52	42	32	18	18	16	8	4	

**Table 3:** Overview of recent LCA studies of biocomposites.

Biocomposite	Conventional Counterpart	System Boundaries	Main LCA Finding	Hotspots	References
	FOSSIL-BASED				
PLA/hemp	PET	cradle-to-gate	30–44% higher environmental impacts. The improved model showed 57% lower GWP and 43% lower smog formation than a virgin PET, while outperforming it in fossil fuel depletion	Harvesting stage, linked to the use of biomass, fertilisers, and diesel fuels	[83]
PHBV:PBAT/rice straw	PP, PET, LDPE, PVC	cradle-to-grave	Lower or comparable climate change potential, however, impacts in other categories are 2–5 times higher, mainly due to long transport distances, inclusion of cultivation-related processes, and energy-intensive laboratory-scale processing	PHBV and PBAT production	[66]
PLA/hemp/silver nanoparticles (AgNP)	PP, HDPE	cradle-to-grave	Higher environmental impacts. Impacts could be mitigated through process optimisation and energy reduction strategies.	Energy demand, particularly from AgNP synthesis	[68]
Cellulose acetate/miscanthus fibre	PP/GF	cradle-to-gate	Higher environmental impacts	Cellulose acetate production, especially acetic anhydride use	[50]
PLA/hemp and PLA/flax	PA66/GF	cradle-to-gate	Lower GWP, but higher impact in eutrophication, acidification, and terrestrial ecotoxicity	Crop growing	[58]
PLA:TPS blends/bleached kraft hardwood fibres	PP/GF	cradle-to-gate	Lower environmental impact in carbon footprint, total energy consumed and air acidification, while higher impact on water eutrophication	TPS production	[67]
PLA/wood flour, modified starch/wood flour and PHBV/wood flour	PP/wood flour, LDPE/wood Flour, PVC/wood Flour, HDPE/wood flour	cradle-to-gate	Lower impact on GWP, fossil fuel depletion, and certain human health impacts. Higher environmental impacts in eutrophication	Plastic matrix production and injection moulding	[52]
PHB/hybrid kenaf with oil palm fibres	PP/GF and PE/GF	cradle-to-gate	Human health impacts are reduced by more than 50%	Not specified	[104]
Epoxidized sucrose soyate resin/flax	BPA-based composite	cradle-to-gate	Lower impacts in all categories except ozone depletion and eutrophication	Vegetable oil methyl ester is used for producing sucrose Soyate and cross-linker	[81]
PHBV/vine shoot particles, PLA/vine shoot particles	PP/vine shoot particles, PP/vine shoot particles	cradle-to-grave	Reduced GWP compared to virgin plastic trays. Overall environmental performance is improved across most impact categories	Polymer matrix production and milling for filler	[23]
	BIOPLASTICS				
PLA/potato pulp, PHA/potato pulp	Pure PLA and PHA	cradle-to-gate	Increase GHG emissions, mainly due to thermal drying	Drying of potato pulp	[5]
PLA/olive wood scraps	Pure PLA	cradle-to-gate	Increase in the impacts in the grinding and extrusion phases, while the total impact presents the lower values (between 5.5% and 10% less)	Production of PLA granulate, followed by grinding and drying. Wooden scraps increase the impact in the extrusion and 3D printing stages	[44]
	BIOCOMPOSITES				
PLA/kraft lignin or hemp straw	Different fibres	cradle-to-grave	Hemp straw showed lower environmental impacts	Filament production for biocomposite with kraft lignin, straw pre-processing for hemp	[47]
PLA/durian skin fibre	With and without epoxidized palm oil	cradle-to-grave	Lower environmental impacts due to a lower amount of energy required for production	Injection moulding, extrusion and drying of durian skin fibre in GWP and acidification potential categories, while drying in ozone depletion potential and alkaline treatment in eutrophication	[50]
PBS/coffee grounds	Different fillers range	cradle-to-gate	Ratio increases reduce the whole-system related CED and carbon footprint by 7.4–8.4%	Electricity for drying and injection moulding	[48]
Starch/Agava fibres	Three processes to elaborate biofilms and starch sources	cradle-to-gate	Corn starch and the green single-step method process have the best eco-efficiency, marked with the lowest environmental impacts	Electricity consumption	[102]
PLA/flax fibres and PLA/bamboo fibre	Different fibres	cradle-to-gate	PLA/bamboo biocomposite has a smaller environmental footprint than the PLA/flax biocomposite	Surface treatment of fibres	[45]
PVOH/cellulose	Conventional melt blending technology	gate-to-gate	Lower environmental impacts by 58–92%, the biggest reduction in “Water use” indicator	Granules oven drying	[71]
PLA/bagasse or PLA/banana fibres (raw, NaOH-treated, copper-coated)	Different fibres and their treatment	cradle-to-gate	Copper coating is the best in terms of GWP reduction. Banana composites show lower impact than bagasse.	Electricity for the manufacturing process, mainly for the drying of fibres	[96]
Sago starch/Sugar palm fibre	-	cradle-to-grave	Low overall impacts, with climate change and human health damages dominated by fossil-based CO_2_ emissions from electricity and heat generation	Electricity for compounding, extrusion and thermoforming	[43]

## Data Availability

No new data were created or analyzed in this study. Data sharing is not applicable to this article.

## Abbreviations

The following abbreviations are used in this manuscript:

AgNP	Silver Nanoparticles
Bio-PA 11	Bio-based Polyamide 11
BioPE	Bio-based Polyethylene
BPA	Bisphenol A
CA	Cellulose Acetate
CE	Circular Economy
CFs	Characterisation factors
DPP	Digital Product Passport
EAS	Environmental Assessment Strategies
EoL	End-of-Life
ESPR	Ecodesign for Sustainable Products Regulation
ESS	Epoxidised Sucrose Soyate
FU	Functional unit
GF	Glass Fibre
GHG	Greenhouse gas
GWP	Global Warming Potential
HDPE	High-Density Polyethylene
ISO	International Organisation for Standardisation
LCA	Life Cycle Assessment
LCI	Life Cycle Inventory
LCIA	Life Cycle Impact Assessment
LCSA	Life Cycle Sustainability Assessment
LDPE	Low-Density Polyethylene
MarILCA	Marine Impacts in Life Cycle Assessment
mch-PLAs	Medium-Chain-Length Polyhydroxyalkanoates
NM	No mentioned
PA	Polyamide
P4HB	Poly(4-hydroxybutyrate)
PBAT	Poly(butylene adipate-co-terephthalate)
PBS	Polybutylene Succinate
PCL	Polycaprolactone
PDLA	Poly(D-lactic acid)
PE	Polyethylene
PEF	Product Environmental Footprint
PET	Polyethylene Terephthalate
PHA	Polyhydroxyalkanoates
PHB	Polyhydroxybutyrate
PHBH	Poly(3-hydroxybutyrate-co-3-hydroxyhexanoate)
PHBV	Poly(3-hydroxybutyrate-co-3-hydroxyvalerate)
PLA	Polylactic Acid
PLLA	Poly(L-lactic acid)
PP	Polypropylene
PVC	Polyvinyl Chloride
PVOH	Polyvinyl Alcohol
SC-PLA	Stereocomplex Polylactic Acid
SEA	Strategic Environmental Assessment
SSA	Systematic Sustainability Assessment
SSbD	Safe and Sustainable by Design
TPS	Thermoplastic Starch

## Appendix A

**Table A1:** Comparison of main LCA software.

Software Name	Primary Focus	Licence Type	Key Strengths	Noted Limitations	Typical Users
openLCA	Open-source, comprehensive LCA	Free/Open-source	World’s most widely used; full transparency and flexibility; powerful and modular; supports advanced analysis and collaboration; versatile for all user groups.	Significant learning curve for advanced features; full database access often requires additional fees.	Researchers, Academics, Consultants, Industry, Educators
SimaPro	Expert-level LCA modelling and enterprise scaling	Commercial	Proven reliability; advanced analysis (scenario, uncertainty); comprehensive standards compliance; integrates with high-quality databases; strong support.	Steep learning curve; high licencing fees can be a barrier; manuals may not always be sufficient for specific processes.	Research Institutes, Consultants, Large Firms, Academia
Sphera LCA for Experts (GaBi)	Industrial LCA modelling and reporting	Commercial	Established tool with extensive sector-specific databases; strong compliance with international standards; robust QA; faster calculations with aggregated data.	Aggregated data limits modification; restricted to GaBi software; primarily attributional; not cloud-based.	Industry Experts, Sustainability Consultants
Umberto	LCA Practitioners and Experts	Commercial	Visual Modelling with Sankey Diagrams; Integrated Costing (MFCA), Scenario Analysis: Powerful “what-if” capabilities	“expert-grade” tool with high granularity, often requires more manual setup,	LCA Practitioners and Experts, Sustainability Consultants, Industrial Engineer, Researchers and Students
